# Network topology and entropy analysis of tetragonal farneseite zeolites

**DOI:** 10.1038/s41598-025-90177-y

**Published:** 2025-04-28

**Authors:** Kavin Jacob, Joseph Clement, Micheal Arockiaraj, Pancras Peter, Krishnan Balasubramanian

**Affiliations:** 1 Department of Mathematics, School of Advanced Sciences, Vellore Institute of Technology, Vellore, India; 2 Department of Mathematics, Loyola College, Chennai, India; 3 School of Molecular Sciences, Arizona State University, Tempe, AZ 85287-1604 USA

**Keywords:** Distance-based topologies, Network topology of phase transitions, Sodalites, Zeolite farneseite, Szeged-type entropies, Chemistry, Materials science, Computational science

## Abstract

Topological indices and their entropies of networks of complex materials and chemical structures are vital for quantitative structure activity and property relationship studies. Computing these measures for complex three-dimensional (3D) crystalline frameworks has been challenging owing to their sizes and complexities. Tetragonal zeolite farneseite (FAR) is a mineral from the cancrinite sodalite group with potential applications in the field of microelectronics, medicine, environment, and industry. We have obtained generalized expressions for the various indices and entropies of these complex networks using a graph theoretical cut method to compute distance-based indices for the FAR framework. With the aid of computed indices and Shannon’s entropy formula, we explore the structural complexity of the frameworks. The present study reveals that the bond-wise entropies of sodalite (SOD) frameworks are greater compared to the farneseite-type frameworks which would have significant ramifications on phase transitions and other phenomena pertinent to such complex structures that typically undergo metamorphosis from one structural network topology to another complex network.

## Introduction

Zeolites which are microporous minerals, present a multitude of prospects for utilizing them in chemical processing industries due to their superior physico-chemical characteristics, elevated thermal stability, and their tunability of textural characteristics, such as pores, cavity sizes, and shapes^1,2^. Fluid catalytic cracking (FCC) utilizes zeolites, particularly ZSM-5 and Y-zeolites, to break down large hydrocarbon molecules into gasoline, diesel, and other valuable products^3^. For zeolite materials, the metal-nonmetal bonds exhibit a varied character between ionic and covalent bonding depending on the participating moieties. Furthermore, the ionicity can change depending upon which tetrahedral elements make up the structure. For example, the interactions in aluminophosphate systems can be better comprehended in terms of ionic bonds between Al$^{3+}$ and PO$_4^{3-}$ ions, whereas the chemical bonds in all-silica zeolites exhibit a more covalent character^4,5^.

The interesting part in the study of zeolite frameworks is the characterization of network and bonding complexities via computational techniques. Zeolite frameworks comprised of Si, Al, P, and occasionally other atoms in fully cross-linked tetrahedra are generally called as T-atoms. Zeolite systems exhibit distinctive structural characteristics; they resemble 4-connected nets with T-atoms connecting to nearby neighbors via oxygen bridges. As a result, by employing various mathematical techniques and taking topological factors into account, it is feasible to list periodic 3-dimensional 4-connected nets^6^. Heavy elements such as Pd, W, Ir, Au, Pt, etc., can also be incorporated into the zeolite framework due to their capability to stimulate high catalytic activity. Experimental studies and computer simulations have shown that the kind of non-framework anions present in addition to the micropore volume, particular area of surface, channel size, and structure of zeolites all influence their ability to sorption of hydrogen^7^. Zeolites have the potential to conduct ions, particularly when they are hydrated or in the presence of certain cations such as Na$^+$, K$^+$, or Ca$^{2+}$. This characteristic holds significance in sensor and ion-exchange membrane applications^8^.

**Figure 1:**
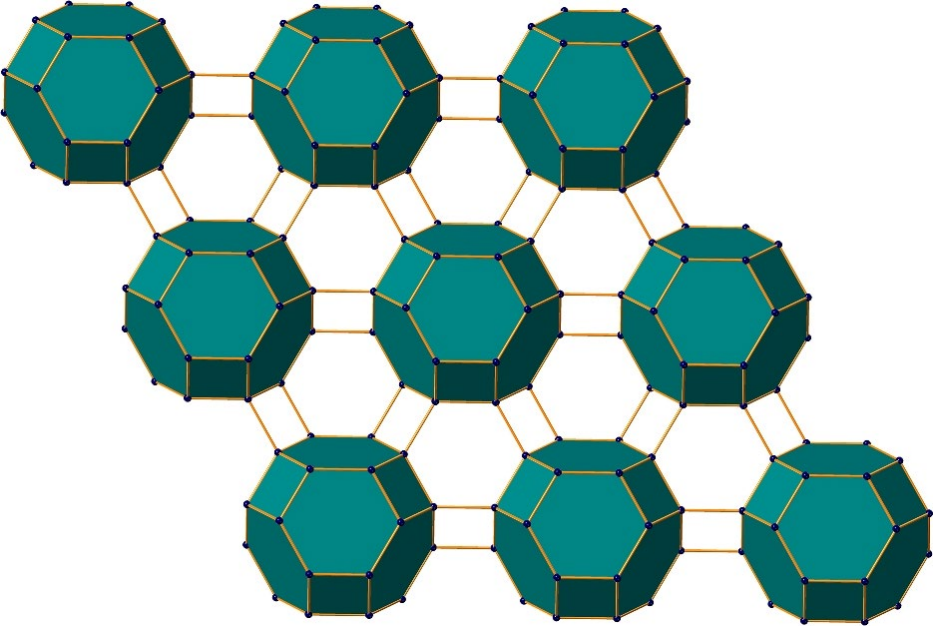
Polyhedral view of zeolite FAR.

Zeolite FAR was originally identified in 2006 in Italy as the structure of farneseite with molecular formula (Na,Ca,K)$_{56}$(Al$_6$Si$_6$O$_{24}$)$_7$(SO$_4$)$_{12}$.6H$_2$O^9^. Its structure is made of TO$_4$ tetrahedra with shared corners, where T is often Si or Al. Zeolite FAR has an hexagonal structure with a cell volume of 4915.6 Å$^3$. It has two types of channels: one composed of 12-membered rings (12MR) and the other contains 8-membered rings (8MR). The 12MR channels have the largest diameter (6.36 Å), while the 8MR channels are elliptical (2.47 Å and 2.26 Å). The framework density of FAR is 17.1 T/1000 Å$^3$, indicating that the material has 17.1 tetrahedral sites (T-sites) per 1000 Å$^3$ within its framework. This measures how densely the atoms are packed in the framework structure itself, excluding any pores or voids. Additionally, the material has a topological density of 0.693, meaning that there are 0.693 nodes per cubic nanometer (nodes/nm$^3$) in its overall topology. This reflects the connectivity and arrangement of structural nodes and linkers within the material’s network^10^. Together, these densities provide insights into the material’s structural compactness and its potential surface area and porosity. The polyhedral view of zeolite FAR is illustrated in Fig. 1. It can also be synthesized with an imidazole acting as an organic structure-directing agent. The resulting structure is also known as zeolite imidazole frameworks (ZIFs) because it contains transition metals such as Zn or Co at the tetrahedral positions rather than Al or Si, and it has more effective thermal and chemical stability than usual zeolites^11^. Recent advances in the structural characterization of zeolites include high-resolution scanning/transmission electron microscopy with aberration correction for atomic-level analysis, solid-state NMR spectroscopy for detailed insights into local environments and catalytic behaviors, and low-dose electron ptychography for three-dimensional structural and compositional analysis. These techniques provide unprecedented detail on zeolite frameworks, including defects and surface properties, enabling the precise tailoring of zeolites for applications in catalysis, adsorption, and separation processes^12–14^.

Graph theory investigates the characteristics of networks, or graphs, which are made up of nodes and edges. The arrangement of nodes (atoms) and edges (bonds) in the zeolite structure is known as the zeolite framework, and graph theory can be used to characterize its topology and provide contrasts for different network arrangements^15,16^. The phase transition phenomena accompanied by structural metamorphosis under the influence of heat or pressure can be characterized using graph theory. A graph theory-based mathematical technique is presented in^17^ to explain the rarity of potential zeolite structures in nature as well as in predicting the combinations of zeolite types that are capable of experiencing these types of structural changes. Graph theory can also be applied to analyze the neighborhood patterns within the zeolite frameworks. They aid in understanding the influence of topology on molecular adsorption and catalysis within the pores of zeolites^18–20^. In a recent study, the role of zeolite structure in stability and catalysis was examined through atomic simulations, using machine learning approaches^21^.

Topological indices, which depend on topological distances between vertices or the degrees of vertices are numerical numbers that quantify topological structural information of networks^22–25^. Quantitative structure-property/activity relationship (QSPAR) investigations are beneficial in that they attempt to predict the physical characteristics or biological activities of chemical compounds and materials^26–29^. The relativistic structural descriptors of zeolite frameworks can potentially incorporate relativistic effects which find applications in characterizing the morphological changes while heavier elements are present in zeolite frameworks^30–35^. Degree and neighborhood degree-based indices along with statistically derived entropy can provide information on networks^36,37^. Combining statistical models such as linear, non-linear, and multivariate regressions along with topological indices provides positive insights into QSPR correlation for the zeolite framework^38–41^.

The main objective of this article is to compute the various distance-based topological indices of zeolite FAR framework by calculating topological distances between all pairs of atoms in its molecular graph. However, this can be challenging with large and complicated zeolite structures, particularly those with tunnels or cages. To derive the various distance-based topological indices of FAR zeolite, in “Graph theoretical techniques” we have presented an efficient cut method to simplify the network and reduce the computational burden of determining the distance-based topological indices. With the aid of the cut method, we have derived generalized expressions to calculate distance based indices in “Topological aspects of zeolite FAR”. The estimation of Szeged-type entropy of the FAR framework along with bond-wise entropy comparison with zeolite SOD framework have been carried out in “Information entropies of FAR”.

## Graph theoretical techniques

In this article, we represent *V*(*G*) and *E*(*G*) as the vertex and edge set of a simple connected graph *G*. The notations |*V*(*G*)| and |*E*(*G*)| represent the cardinality of the vertex and edge set, which is commonly referred to as the size and order of *G*. The distance between a pair of vertices in G is defined as the length of the shortest path between those pairs and it is denoted by $d_G(u,v)$, $u,v\in V(G)$. Let $v\in V(G)$ and $e = ab\in E(G)$ then the distance $d_G(v,e)$ between them is defined as $min(d_G(v,a),d_G(v,b))$. Similarly, we define the distance between any two pairs of edges $e = ab,f = cd\in E(G)$ is defined as the minimum number of edges along a shortest (*e*, *c*)-path or a shortest (*e*, *d*)-path and denoted by $D_G(e,f)$. By considering an edge $e = ab\in E(G)$, we define the following notations which play a crucial part in establishing Szeged and Mostar-type indices.

$$\begin{aligned} n_a(e|G)&= |\{u\in V(G):d_G(a,u)<d_G(b,u)\}| \\ m_a(e|G)&= |\{h\in E(G):d_G(a,h)<d_G(b,h)\}| \end{aligned}$$

In addition, we define $n_b(e|G)$ and $m_b(e|G)$ analogously with respect to *b*. By utilizing the above defined notations, we present the following distance indices of *G*.

1. **Wiener-type indices**
  $$\displaystyle W_v(G) = \sum _{\{u,v\}\subseteq V(G)}{d_G(u,v)}$$, (Wiener)^42^
  $$\displaystyle W_e(G) = \sum _{\{e,f\}\subseteq E(G)}{d_G(e,f)}$$, (Edge Wiener)^43^
  $\displaystyle W_{ve}(G) = \frac{1}{2} \sum _{u\in V(G)}\sum _{e\in E(G)}{d_G(u,e)}$, (Vertex-edge Wiener)^43^
2. **Szeged-type indices**
  $$\displaystyle SZ_v(G) = \sum _{e=ab\in E(G)}{n_a(e|G)n_b(e|G)}$$, (Vertex Szeged)^44^
  $$\displaystyle SZ_e(G) = \sum _{e=ab\in E(G)}{m_a(e|G)m_b(e|G)}$$, (Edge Szeged)^45^
  $\displaystyle SZ_{ve}(G) = \frac{1}{2} \sum _{e=ab\in E(G)}{n_a(e|G)m_b(e|G)+n_b(e|G)m_a(e|G)}$, (Vertex-edge Szeged)^46^
  $\displaystyle PI_{v}(G) = \sum _{e=ab\in E(G)}{n_a(e|G)+n_b(e|G)}$, (Vertex Padmakar-Ivan)^47^
  $\displaystyle PI_{e}(G) = \sum _{e=ab\in E(G)}{m_a(e|G)+m_b(e|G)}$, (Edge Padmakar-Ivan)^47^
3. **Mostar-type indices**
  $$\displaystyle MO_{v}(G) = \sum _{e=ab\in E(G)}{|n_a(e|G)-n_b(e|G)|}$$, (Vertex Mostar)^48^
  $$\displaystyle MO_{e}(G) = \sum _{e=ab\in E(G)}{|m_a(e|G)-m_b(e|G)|}$$, (Edge Mostar)^49^

In “Partial cubes and cut method”, we elaborate the concepts of cut method which is an efficient tool in computing these indices.

### Partial cubes and cut method

For the edge set of a graph *G*, we define Djoković-Winkler relation $\Theta$ as follows: Two edges $e,f\in E(G)$ are said to have $\Theta$ relation i.e, $e\Theta f$ if $d_G(a,c)+d_G(b,d)\ne d_G(a,d)+d_G(b,c)$^50,51^. This $\Theta$ relation plays a major role on defining the characteristics of a partial cube. A graph *G* is a partial cube, we see that $\Theta$ relation is always a equivalence relation and removal of each $\Theta$ class from *G* precisely leaves two components.

Let *G* be a partial cube, and $H \subseteq E(G)$ be a $\Theta$ class. Then the quotient graph *G*/*H* is a path of two vertices $P_2$, whose vertices represents the connected components of $G-H$^51,52^. We state the following theorem to find the distance based topological indices using the properties of partial cube.

#### Theorem 2.1

*Let G be a partial cube with* $\Theta$ *partitions* $\mathscr {P} = \{\alpha _1,\alpha _2,...\alpha _l\}$. *For each* $\alpha _i$, $1\le i\le l$, *the quotient graph* $G/\alpha _i$ *is a path with two vertices which represents the connected components* $G_{1i}$ *and* $G_{2i}$. *We define* $n_1(\alpha _i)= |V(G_{1i})|$, $m_1(\alpha _i)= |E(G_{1i})|$, $n_2(\alpha _i)= |V(G_{2i})|$ *and* $m_2(\alpha _i)= |E(G_{2i})|$. *Then*,

1. $$\displaystyle W_v(G)= \sum _{i=1}^l n_1(\alpha _i)n_2(\alpha _i)$$
2. $$\displaystyle W_e(G)= \sum _{i=1}^l m_1(\alpha _i)m_2(\alpha _i)$$
3. $$\displaystyle W_{ve}(G)= \frac{1}{2} \sum _{i=1}^l n_1(\alpha _i)m_2(\alpha _i) + n_2(\alpha _i)m_1(\alpha _i)$$
4. $$\displaystyle SZ_{v}(G)= \sum _{i=1}^l |\alpha _i| \ n_1(\alpha _i)n_2(\alpha _i)$$
5. $$\displaystyle SZ_{e}(G)= \sum _{i=1}^l |\alpha _i| \ m_1(\alpha _i)m_2(\alpha _i)$$
6. $$\displaystyle SZ_{ve}(G)= \frac{1}{2} \sum _{i=1}^l \ |\alpha _i| \ (n_1(\alpha _i)m_2(\alpha _i) + n_2(\alpha _i)m_1(\alpha _i))$$
7. $$\displaystyle PI_{v}(G)= \sum _{i=1}^l |\alpha _i| \ ( n_1(\alpha _i) + n_2(\alpha _i) )$$
8. $$\displaystyle PI_{e}(G)= \sum _{i=1}^l |\alpha _i| \ ( m_1(\alpha _i) + m_2(\alpha _i) )$$
9. $$\displaystyle MO_{v}(G)= \sum _{i=1}^l |\alpha _i| \ | n_1(\alpha _i) - n_2(\alpha _i) |$$
10. $$\displaystyle MO_{e}(G)= \sum _{i=1}^l |\alpha _i| \ | m_1(\alpha _i) - m_2(\alpha _i) |$$

By using Theorem 2.1, we derive the distance based descriptors of zeolite farneseite network in forthcoming section.

## Topological aspects of zeolite FAR

**Figure 2:**
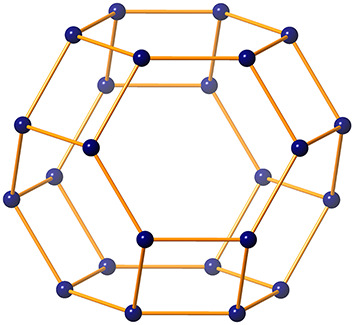
Unit cell of zeolite FAR.

The crystal lattice of zeolite farneseite is constructed by tiling the 3D unit cell in $p \times q$ mesh, where *p* and *q* represent the number of rows and columns, respectively arranged to form the structure of *FAR*. The unit cell consists of 24 atoms and 36 bonds, in which 6 hexagons and 6 squares are connected by common edges as shown in Fig. 2.

**Figure 3:**
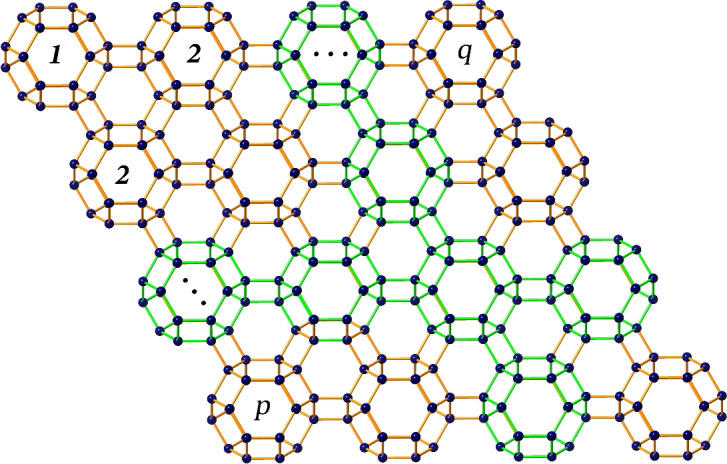
Schematic growth of *FAR*(*p*, *q*).

**Figure 4:**
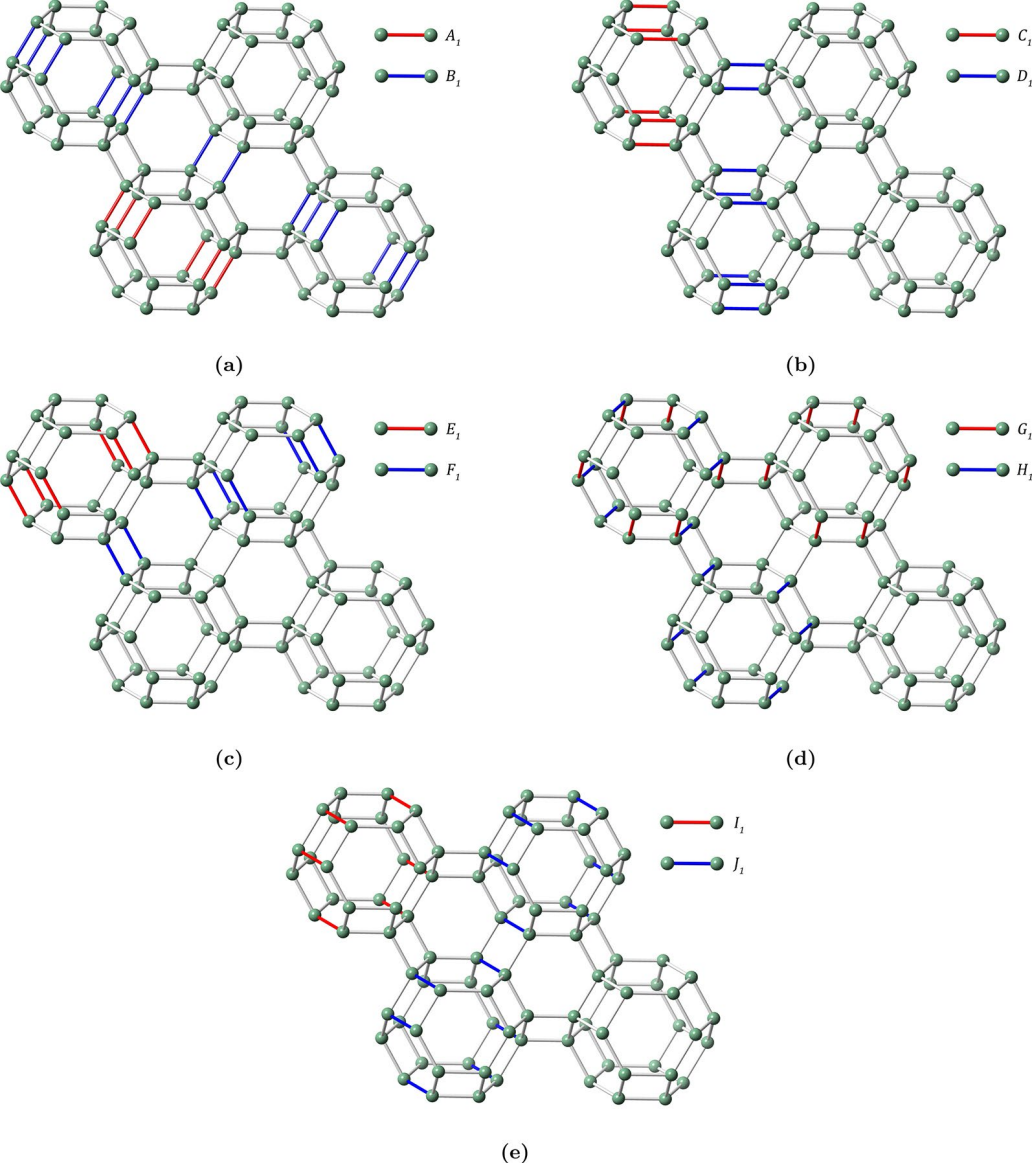
Illustration of various $\Theta$ partitions of *FAR*(2, 2).

Let us denote the lattice of zeolite farneseite as *FAR*(*p*, *q*), where the front view represents the schematic growth along the *p* and *q* axes as illustrated in Fig. 3. Here, we employ translational symmetry of the unit cell with the parameters *p* and *q* to capture the essential structural features of the zeolite framework. This approach balances computational efficiency with accurate representation, ensuring that significant topological properties are preserved. In zeolite *FAR*, the coordinates *p* and *q* are isomorphic to a cyclic permutation i.e. $FAR(p,q) \cong FAR(q,p)$. The total number of atoms and bonds in *FAR*(*p*, *q*) are denoted by $|V(FAR(p,q))|= 24pq$ and $|E(FAR(p,q))|= 2(21pq-2p-2q+1)$. A carefull scrutiny of the molecular graph of *FAR* with the aid of Djoković-Winkler relation and its properties suggests that the FAR zeolite can be characterized by a partial cube. Therefore, we classify $\Theta$ partition set into 6 varieties of cuts such as obtuse $\{A_i,A_i^{\prime},B_i\}$, vertical $\{C_i,C_i^{\prime},D_i\}$, acute $\{E_i,E_i^{\prime},F_i\}$, side-obtuse $\{G_i\}$, top-obtuse $\{H_i\}$ and adjusted side-acute cuts $\{I_i,I_i^{\prime},J_i\}$, that unions of these 6 cut sets results in *E*(*FAR*(*p*, *q*)). Here we observe that $\{A_i^{\prime},C_i^{\prime},E_i^{\prime},I_i^{\prime}\}$ cuts are symmetric to $\{A_i,C_i,E_i,I_i\}$. Graphical representation of these cuts are shown in Fig. 4.

**Figure 5:**
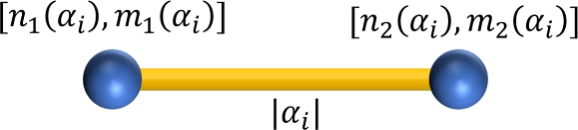
Quotient graph $$G/\alpha _i$$.

Now, we classify the graph theoretical parameters of the standard cut method to derive generalized expressions for computing the distance-based indices of the farneseite framework. We consider $2 \le p \le q$, in order to generalize the cuts of *FAR*(*p*, *q*). It is sufficient to swap out *p* and *q* in the case $p \ge q$ since the molecular graph *FAR*(*p*, *q*) is isomorphic to *FAR*(*q*, *p*). The quotient graph $G/\alpha _i$ obtained for each class of $\Theta$ partition is a $P_2$ graph with vertex parameters $[n_1(\alpha _i),m_1(\alpha _i)]$ and $[n_2(\alpha _i),m_2(\alpha _i)]$ along with edge cardinality parameter $|\alpha _i|$ as illustrated in Fig. 5. The generalized parameters of $\{A_i,A_i^{\prime},B_i,C_i,C_i^{\prime},D_i,G_i,H_i,I_i,I_i^{\prime},J_i\}$ cuts are presented in Table 1.

**Table 1:** Parameters associated with quotient graphs of $\Theta$ classes.

$\alpha _i$	Range	$n_1(\alpha _i)$	$m_1(\alpha _i)$	$|\alpha _i|$
$A_i,A_i^{\prime}$	$1\le i\le p-1$	$12i^2$	$21i^2 - 8i + 2$	$8i-2$
$B_i$	$1\le i\le q-p+1$	$12p(p + 2i - 2)$	$42pi - 4i - 50p + 21p^2 + 6$	$8p-2$
$C_i,C_i^{\prime}$	$1\le i\le p-1$	$6i(i+1)$	$\frac{1}{2} (11i + (-1)^i + 21i^2 - 1)$	$4i - (-1)^i + 1$
$D_i$	$1\le i\le 2q - p$	$6p(p + 2i - 1)$	$\frac{1}{2} (42pi - 4i - 31p + 21p^2 + (-1)^i - (-1)^{p+i} + 4)$	$4p - (-1)^i + (-1)^{p+i}$
$G_i$	$1\le i\le p$	$12q(2i-1)$	$16q + (42q - 4)(i - 1) - 1$	6*q*
$H_i$	$1\le i\le q$	$12p(2i-1)$	$16p + (42p - 4)(i - 1) - 1$	6*p*
$I_i,I_i^{\prime}$	$1\le i\le p-1$	$12i^2$	$21i^2 - 7i + 1$	6*i*
$J_i$	$1\le i\le q-p+1$	$12p(p + 2i - 2)$	$42pi - 4i - 49p + 21p^2 + 5$	6*p*

While generalizing the parameters of $E_i$, $E_i^{\prime}$ and $F_i$ cuts, the problem is divided into 2 cases i.e, $2p-1<q$ and $2p-1\ge q$. The parameters of these classes are listed in Table 2.

**Table 2:** Parameters associated with quotient graphs of acute $\Theta$ classes.

$\alpha _i$	Case	Range	$n_1(\alpha _i)$	$m_1(\alpha _i)$	$|\alpha _i|$
$E_i,E_i^{\prime}$	$2p-1<q$	$1\le i\le 2p-2$	$6i(i+1)$	$\frac{1}{2}(11i + (-1)^i + 21i^2 - 1)$	$4i - (-1)^i + 1$
$E_i,E_i^{\prime}$	$2p-1\ge q$	$1\le i\le q-1$	$6i(i+1)$	$\frac{1}{2}(11i + (-1)^i + 21i^2 - 1)$	$4i - (-1)^i + 1$
$F_i$	$2p-1<q$	$1\le i\le q-2p+2$	$12p(2p + 2i - 3 )$	$42pi - 4i - 73p + 42p^2 + 8$	$8p - 2$
$F_i$	$2p-1\ge q$	$1\le i\le 2p-q$	$6q(q + 2i - 1)$	$\frac{1}{2}(42qi - 4i - 31q + 21q^2 + (-1)^i - (-1)^{q+i} + 4)$	$4q - (-1)^i + (-1)^{q+i}$

The remaining vertex parameters are given by the formula $n_2(\alpha _i) = |V(G)|-n_1(\alpha _i)$ and $m_2(\alpha _i) = |E(G)|-m_1(\alpha _i)-|\alpha _i|$. Let $X \in \{ W_v, W_e, W_{ve}, SZ_{v}, SZ_{e}, SZ_{ve}, PI_{v}, PI_{e}, MO_{v}, MO_{e} \}$, then the generalized expression to evaluate distance based indices of *G* is given as:

1 $$\begin{aligned} X(G) = \sum _{i=1}^{l}X(G/\alpha _i) \end{aligned}$$

### Case I:$2p-1<q$

We expand Eq. (1) by incorporating the parameters of each $\Theta$ classes from Tables 1 and 2 to derive the following generalized expression to evaluate distance based indices of *FAR*. Then,

2 $$\begin{aligned} X(G) =&2\sum _{i=1}^{p-1}X(G/A_i)+\sum _{i=1}^{q-p+1}X(G/B_i)+2\sum _{i=1}^{p-1}X(G/C_i)+\sum _{i=1}^{2q-p}X(G/D_i)+2\sum _{i=1}^{2p-2}X(G/E_i)+ \nonumber \\&\sum _{i=1}^{q-2p+2}X(G/F_i)+\sum _{i=1}^{p}X(G/G_i)+\sum _{i=1}^{q}X(G/H_i)+2\sum _{i=1}^{p-1}X(G/I_i)+\sum _{i=1}^{q-p+1}X(G/J_i) \end{aligned}$$

By substituting the parameter values and indices formula form Theorem 2.1 in Eq. (2) we obtain the following theorem:

#### Theorem 3.1

Let *G* be molecular graph of FAR and $2p-1<q$. Then the distance based indices of *G* are

1. $$W_{v}(G) = \frac{1}{5}\big [-492p^5 + 1560p^4q + (480q^2 + 540)p^3 + (2880q^3 - 840q)p^2 + (240q^2 - 48)p\big ]$$
2. $$W_{e}(G) = \frac{1}{60}\big [-18081 p^{5}+(57330 q+3150) p^{4}+(17640 q^{2}-25200 q+15625) p^{3}+(105840 q^{3}$$$$-88200 q^{2}+8850 q-2010) p^{2}+(-20160 q^{3}+37680 q^{2}-13080 q+1406) p+960 q^{3}-1920 q^{2}+1380 q$$$$-270+30 (-1)^{p} (p-2 q-1)\big ]$$
3. $$W_{ve}(G) = \frac{1}{5}$$ $$\big [-861 p^{5}-(-2730 q-75) p^{4}-(-840 q^{2}+600 q-855) p^{3}$$ $$-(-5040 q^{3}+2100 q^{2}+810 q+15)$$ $$p^{2}-(480 q^{3}-900 q^{2}+120 q+54) p\big ]$$
4. $$SZ_{v}(G)= \frac{1}{5}\big [-1368 p^{5}-(-960 q+660)$$$$p^{4}-(-20160 q^{3}-3600 q+120) p^{3}$$ $$-(1920 q^{3}-960 q-2100) p^{2}-(-1440 q^{3}-360 q-48)$$$$p+90+(180 p^{2}-360 q p-90) (-1)^{p}\big ]$$
5. $$SZ_{e}(G)= \frac{1}{120} \big [-95844 p^{5}-(119730-309120 q) p^{4}-(-1481760 q^{3}+987840 q^{2}-352520 q-72140) p^{3}-$$$$S(534240 q^{3}-517920 q^{2}+306240 q-180210) p^{2}$$$$-(-162240 q^{3}+114240 q^{2}-79780 q+41336) p+6945$$$$-5800 q-1280 q^{3}+4800 q^{2}-15 (336 p^{2} q-672 p \,q^{2}-878 p^{2}+1388 q p+64 q^{2}+256 p-152 q+399) (-1)^{p}\big ]$$
6. $$SZ_{ve}(G)= \frac{1}{10}\big [-4676 p^{5}-(-9040 q+2860) p^{4}-(-70560 q^{3}+23520 q^{2}-12320 q-660) p^{3}-$$$$(16080 q^{3}-9840 q^{2}+3520 q-7420) p^{2}-$$$$(-5680 q^{3}+1680 q^{2}-1320 q+604) p-60 q+$$$$300-60 (2 p^{2} q-4 p \,q^{2}-10 p^{2}+18 q p+p-q+5)(-1)^{p}\big ]$$
7. $$PI_{v}(G) = 48pq\big [21pq - 2p - 2q + 1\big ]$$
8. $$PI_{e}(G) =\frac{1}{3}\big [244 p^{3}+(5292 q^{2}-1704 q-24) p^{2}+(-1116 q^{2}+792 q-214) p-84 q+18+48 q^{2}-6 (-1)^{p} (p-2 q-1)\big ]$$
9. $$MO_{v}(G) = 2\big [-12 p^{4}-(-48 q+8) p^{3}-(-204 q^{2}+6 (-1)^{p}-42 (-1)^{q}+42 (-1)^{p+q}+48 q+48) p^{2}-(-12 (-1)^{p} q+$$$$24 q^{2}+6 (-1)^{q}-6 (-1)^{p+q}-12 q+4) p-(-18 q^{2}-3) (-1)^{p}-18 q^{2}-3\big ] + \displaystyle \sum _{i=1}^{2q - p} |(4 p-(-1)^{i}+(-1)^{p+i}) (p-2 q+2 i-1)12 p|$$
10. $$MO_{e}(G) = \frac{1}{6}\big [-252 p^{4}-(-1008 q+224) p^{3}$$$$-(-4284 q^{2}+882 (-1)^{p+q}+126 (-1)^{p}-882 (-1)^{q}+1176 q+1044) p^{2}-$$$$(-210 (-1)^{p+q}+840 q^{2}-348 q-152+(-252 q-12) (-1)^{p}+210 (-1)^{q}) p-12 (-1)^{p+q}$$$$-330 q^{2}+12 q-57-(-378 q^{2}+60 q-57) (-1)^{p}+12 (-1)^{q}\big ]+$$$$\displaystyle \sum _{i=1}^{2q - p} | (4 p -(-1)^{i}+(-1)^{p +i}) (p -2 q +2 i -1)(21 p -2)|$$

### Case II: $2p-1 \ge q$

In Eq. (2), the parameters of the $E_i$ and $F_i$ cuts are replaced with the values provided in Table 2. Moreover, we apply the same method as in the previous section to obtain the following theorem, which computes the distance based indices of zeolite *FAR* when $2p-1 \ge q$.

#### Theorem 3.2

Let *G* be molecular graph of FAR and $2p-1 \ge q$. Then the distance based indices of *G* are

1. $$W_{v}(G) = \frac{12}{5}\big [-9 p^{5}+50 p^{4} q-(-120 q^{2}-5) p^{3}-(-200 q^{3}+10 q) p^{2}-(-10 q^{4}+10 q^{2}-4) p-q^{5}+5 q^{3}-4 q\big ]$$$$-q^{5}+5 q^{3}-4 q\big ]$$
2. $$W_{e}(G) = \frac{1}{60} \big [-3969 p^{5}-(-22050 q+210) p^{4}-(-52920 q^{2}+18480 q-2145) p^{3}-(-88200 q^{3}$$$$+93240 q^{2}-29070 q+1890) p^{2}-(-4410 q^{4}+18480 q^{3}-27570 q^{2}+13200 q-4074) p+46 q-441 q^{5}$$$$-210 q^{4}+2645 q^{3}-1890 q^{2}-240-(-30 p+60 q+30) (-1)^{p}-(60 p-30 q+30) (-1)^{q}\big ]$$
3. $$W_{ve}(G) = \frac{1}{5} \big [-189 p^{5}-(-1050 q +5) p^{4}-$$$$(-2520 q^{2}+440 q -95) p^{3}-(-4200 q^{3}+2220 q^{2}-330 q -5) p^{2}$$$$-(-210 q^{4}+440 q^{3}-330 q^{2}+140 q -94) p -21 q^{5}-5 q^{4}+95 q^{3}+5 q^{2}-74 q \big ]$$
4. $$SZ_{v}(G)= \frac{1}{5} \big [(168 p^{5}+(-960 q -1620) p^{4}+(20160 q^{3}+4560 q -120) p^{3}+(-960 q^{3}+480 q +1620) p^{2}+(-480 q^{4}+$$$$1200 q^{3}+480 q^{2}+720 q -48) p +72 q^{5}+60 q^{4}-120 q^{3}-60 q^{2}+48 q +180) +(180 p^{2}-360 p q -90) (-1)^{p}$$$$-360 (-1)^{q} p q +180 (-1)^{q} q^{2}-90 (-1)^{q}\big ]$$
5. $$SZ_{e}(G)= \frac{1}{120} \big [6300 p^{5}+(60480 q-143250) p^{4}+(1481760 q^{3}-799680 q^{2}+475240 q+20620) p^{3}$$$$+(-571200 q^{3}+409440 q^{2}-275760 q+148770) p^{2}+(-11760 q^{4}+190440 q^{3}$$$$-97680 q^{2}+87320 q-38320) p+3948 q^{5}-850 q^{4}-10740 q^{3}+6370 q^{2}-4608 q+12930+((-5040 q+13170) p^{2}$$$$+(10080 q^{2}-20820 q-3840) p-960 q^{2}+2280 q-5985) (-1)^{p}+15 (672 p^{2} q-336 q^{2} p-64 p^{2}$$$$-1388 p q+878 q^{2}+152 p-256 q-399) (-1)^{q}\big ]$$
6. $$SZ_{ve}(G)= \frac{1}{10} \big [444 p^{5}+(-240 q -5740)$$$$p^{4}+(70560 q^{3}-19040 q^{2}+17440 q +180) p^{3}$$$$+(-15280 q^{3}+7440 q^{2}-4160 q +5740) p^{2}+(-1120 q^{4}+5680 q^{3}-480 q^{2}+2320 q -744) p$$$$+220 q^{5}+140 q^{4}-380 q^{3}-140 q^{2}+40 q +600+((-120 q +600) p^{2}+$$$$(240 q^{2}-1080 q -60) p +60 q -300)$$$$(-1)^{p}+60 (-1)^{q} (4 p^{2} q -2pq^{2}-18 p q +10 q^{2}+p -q -5)\big ]$$
7. $$PI_{v}(G) = 48pq\big [21pq - 2p - 2q + 1\big ]$$
8. $$PI_{e}(G) = \frac{1}{3}\big [116p^{3}+(5292 q^{2}-1512 q+72) p^{2}+(-1212 q^{2}+$$$$696 q-194) p+16 q^{3}+72 q^{2}-94 q+12+(-6 p+12 q+6) (-1)^{p}+(12p-6q+6) (-1)^{q}\big ]$$
9. $$MO_{v}(G) = 2\big [-12 p^{4}-(-48 q-8) p^{3}-(-156 q^{2}+42 (-1)^{p+q}+$$$$9 (-1)^{2 p}+6 (-1)^{p}-18 (-1)^{q}+48 q+39) p^{2}-(-48 q^{3}-12 (-1)^{p} q-12 (-1)^{q} q+$$$$36 q^{2}-6 (-1)^{p+q}+2) p-(-18 q^{2}-3) (-1)^{p}-(6 q^{2}-3) (-1)^{q}-12 q^{4}+4 q^{3}-6 q^{2}-4 q-6\big ]+$$$$\displaystyle \sum _{i=1}^{2q - p} | (4 p-(-1)^{i}+(-1)^{p+i}) (p-2 q+2 i-1)12 p|+$$$$\displaystyle \sum _{i=1}^{2p - q} | (4 q-(-1)^{i}+(-1)^{q+i}) (q-2 p+2 i-1)12 q|$$
10. $$MO_{e}(G) = \frac{1}{6}\big [-252 p^{4}-(-1008 q -112) p^{3}-(-3276 q^{2}+$$$$882 (-1)^{p +q}+126 (-1)^{p}-378 (-1)^{q}+1176 q +996)$$ $$p^{2}-(-210 (-1)^{p +q}+(-252 q -12) (-1)^{p}+(-252 q +60)$$$$(-1)^{q}-1008 q^{3}+1092 q^{2}-144 q -122) p -$$$$12 (-1)^{p +q}-(-378 q^{2}+60 q -57) (-1)^{p}-$$$$(126 q^{2}-12 q -57) (-1)^{q}-252 q^{4}+84 q^{3}-114 q^{2}-60 q -102\big ]+$$$$\displaystyle \sum _{i=1}^{2q - p} | (4 p -(-1)^{i}+(-1)^{p +i}) (p -2 q +2 i -1)(21 p -2)|$$$$+\displaystyle \sum _{i=1}^{2p - q} | (4 q -(-1)^{i}+(-1)^{q +i}) (q -2 p +2 i -1)(21 q -2)|$$

### Numerical and graphical analysis of descriptors

Using the generalized formulas derived in “Topological aspects of zeolite FAR”, we compute the numerical data presented in this section. For the purpose of our investigation, we have chosen the equal case $p=q$ dimensions of the farneseite framework where the unit cell is organized in equal rows and columns. Furthermore, the square dimension $(p=q)$ simplifies the analysis and ensures consistency for a critical comparison of entropy values across the lattice. This approach avoids introducing variable sizes that might arise from non-square dimensions, and thus allows for a more uniform comparison of results. The numerical values of selected distance-based indices for square FAR are listed in Table 3.

**Table 3:** Numerical quantities of distance based indices, when $p=q$.

*X*	*p* values					
	2	3	4	5	6	7
$W_v$	28224	215136	907776	2772000	6899904	14916384
$W_e$	59381	509804	2288719	7259062	18536801	40817452
$W_{ve}$	41040	331608	1442544	4488120	11313600	24681720
$SZ_{v}$	260352	2911104	16303104	62206272	185891328	469192320
$SZ_{e}$	547748	6908128	41208584	163285628	500580420	1286649736
$SZ_{ve}$	378432	4489296	25936128	100825968	305136384	777141744
$PI_{v}$	14784	76896	246528	607200	1266624	2356704
$PI_{e}$	22256	121776	400408	1001000	2109056	3952032
$MO_{v}$	6528	34464	118272	289536	618624	1146912
$MO_{e}$	10784	58008	201056	495040	1061888	1974136

**Figure 6:**
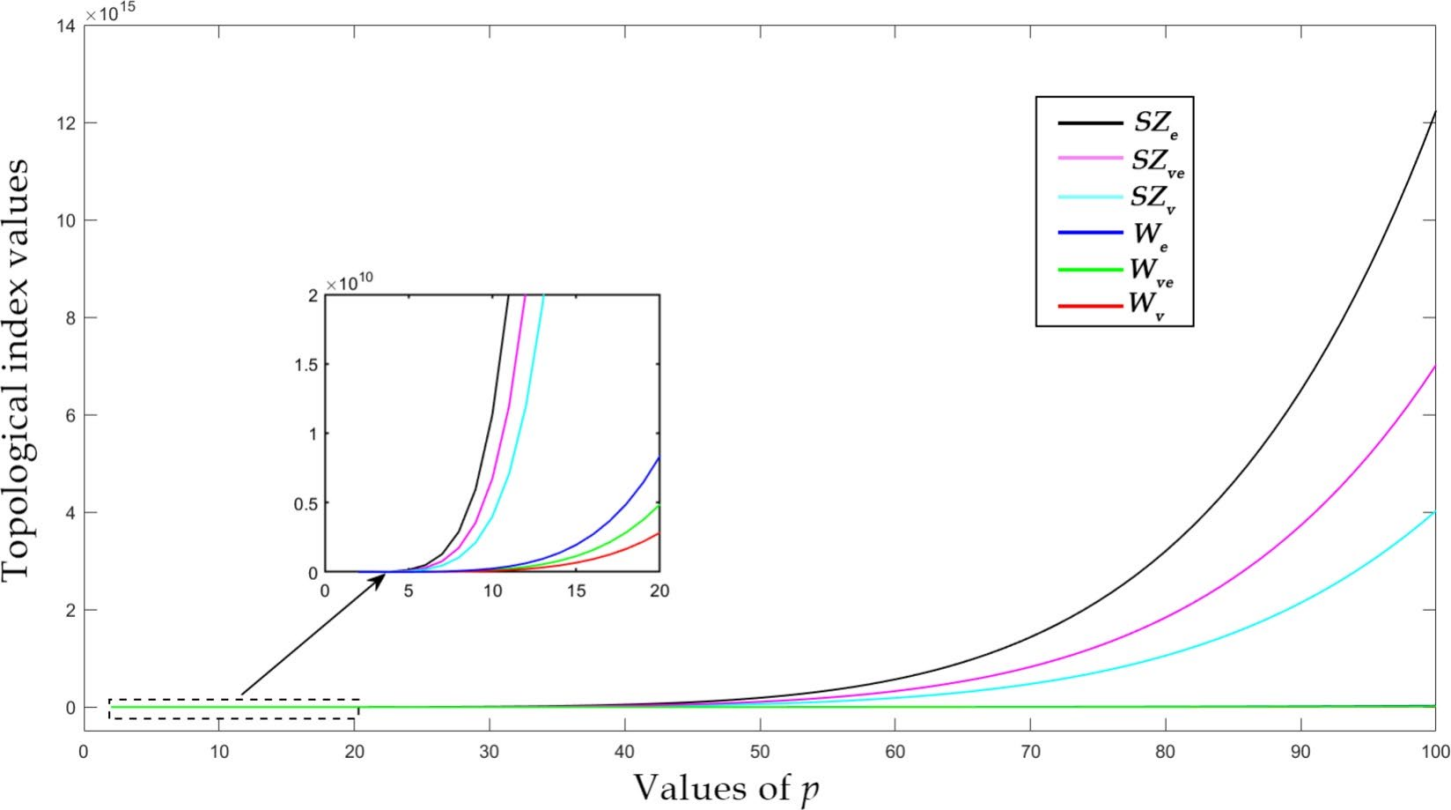
$W_v$, $W_e$, $W_{ve}$, $SZ_v$, $SZ_e$ and $SZ_{ve}$ of square *FAR*.

**Figure 7:**
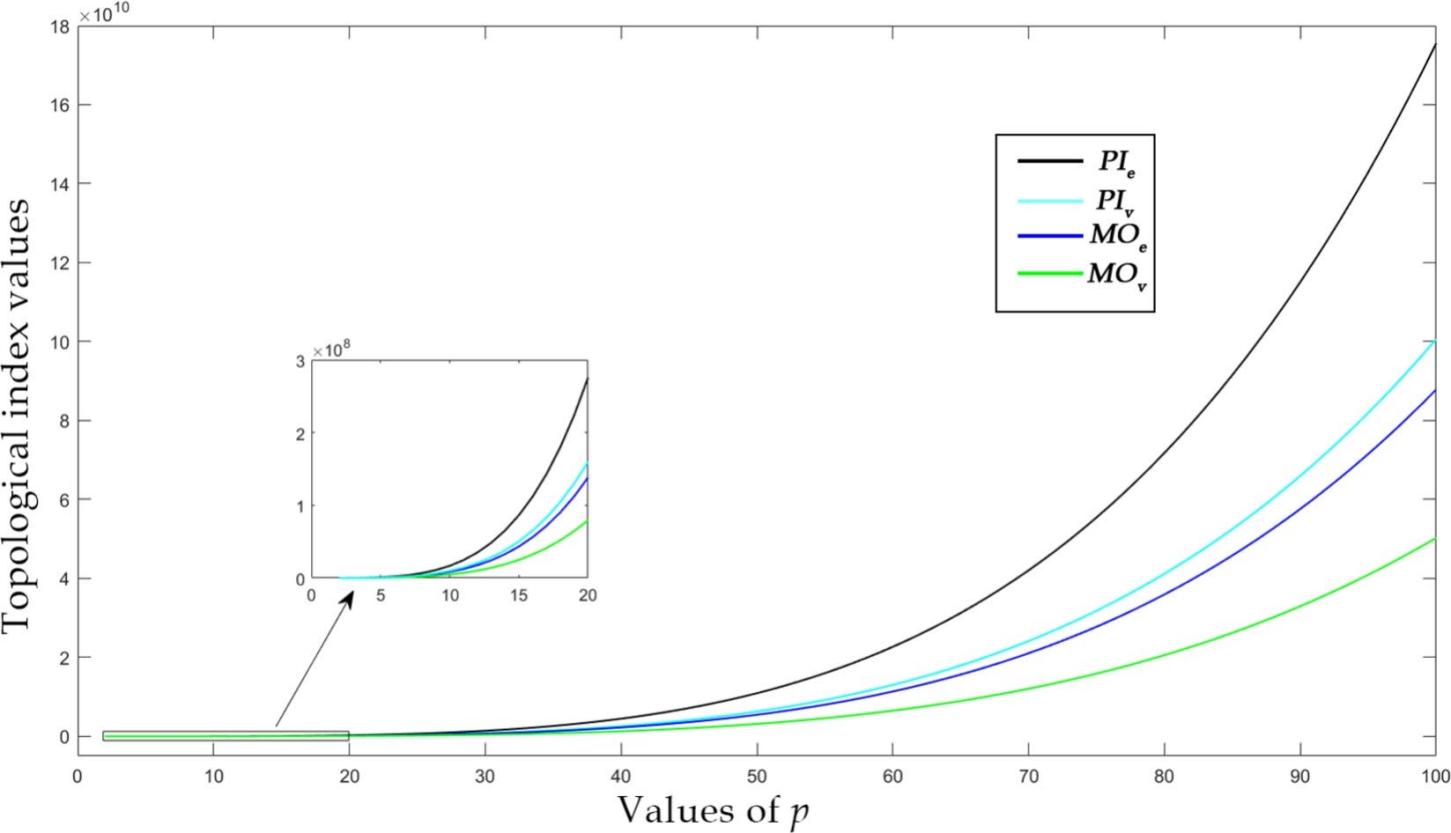
$PI_v$, $PI_e$, $MO_v$ and $MO_e$ indices of square *FAR*.

For graphical visualization, we have incorporated a large range of molecular systems to report a detailed inference of distance-based indices over the farneseite framework. In Figs. 6 and 7, the range of *p* is considered as $2 \le p \le 100$, which implies that the number of atom ranges from 96 to 240,000. Edge version of Szeged index yields the highest value which is illustrated in Fig. 6. On the other hand, Mostar index yields the lowest value as shown in Fig. 7. The Szeged-type indices have the highest values because their contribution is in product form, whereas Mostar-type indices contribute in difference forms. Numerical and graphical analysis of the derived distance based indices of FAR framework is carried out with the aid of MATLAB software. The topological indices and entropies derived from the analytical expressions were compared with the results obtained from TopoChemie-2020^53^ and the results were in complete agreement in all cases.

## Information entropies of FAR

Graph entropy values are significant metrics that measure the order/disorder complexities and can also be interpreted as an information-theoretic index of a graph^54–56^. For researchers to investigate the complexity of the networks, these measures are extremely important in the fields of chemistry, biology, information theory, and sociology. Communication theory had a major impact when Shannon introduced the idea of information entropy to explain the uncertainty in information sources^57^. Shannon defined entropy in terms of the probabilistic measure of discrete random variables^58^. Later, these measures were interpreted in terms of the edge distribution of graphs to find the complexity of graphs^59^. Entropy measures of chemical graphs have been a topic of research in recent years because of their potential applications in QSAPR research^60–63^. Degree-based entropies utilize edge function which is defined in terms of the degree of end vertices of an edge in a graph and has potential applications^36,38–40^. In this section, we determine the distanced-based entropies of the zeolite FAR framework using Shannon’s approach. These measures can be defined for topological indices, such as the Szeged-type indices. Since the Wiener index is not defined in terms of edge function, information entropies are not applicable in its direct form^64^. We exclude the Mostar-type because it is evaluated as indeterminant due to its formula that nullifies the edge function in certain cases. Moreover the Mostar index can become zero for regular graphs resulting in undefined functions for entropies. Entropy-based on topological distance indices, incorporates structural features by considering the various network distances from a given vertex, thereby offering a more comprehensive characterization of the graph’s topology^65^. That is most of the distance-based indices are computed from the distance matrix which contains the shortest distances from any vertex to any other vertex in the molecular network. In contrast, degree-based entropy is limited to local connectivity, focusing solely on the number of edges incident to each vertex. Hence it does not capture the breadth and spread of the overall network structure of the graph. Although the computation of distance-based entropy is more complex due to the need to evaluate topological indices derived from distance matrices, it provides a more detailed and nuanced understanding of the network’s structural properties, particularly for the current molecular networks that we have studied. The graph entropy measure using the topological edge function of a graph *G*^66^ is defined as:

3 $$\begin{aligned} I(X(G)) = \log (X(G)) - \frac{1}{X(G)}\sum _{e\in E(G)} X(e/G) \log (X(e/G)) \end{aligned}$$

In Eq. (3), *X*(*e*/*G*) represents the structural function of an edge $e = ab$ depending on the distance parameters such as {$n_a(e|G),n_b(e|G),m_a(e|G),m_b(e|G)$} associated with the topological index formula. We modify the above equation with respect to cuts defined in Theorem 2.1 to compute the distance based entropies of a partial cube:

4 $$\begin{aligned} I(X(G)) = \log (X(G)) - \frac{1}{X(G)}\sum _{i=1}^{l} |\alpha _i| X(e/\alpha _i) \log (X(e/\alpha _i)) \end{aligned}$$

Here, $X(e/\alpha _i)$ represents topological structural function of an edge in the $\Theta$ class partition $\alpha _i$. By substituting the topological index value obtained from Theorems 3.1 and 3.2 along with the parameters of quotient graph from Tables 1 and 2 in Eq. (4) we obtain the required entropy measure. The Szeged and Padmakar-Ivan-type entropies of square *FAR* have been calculated using Eq. (4) and presented in Table 4.

**Table 4:** Szeged-type entropies of zeolite FAR, $p=q$.

*I*(*X*)	*p* values					
	2	3	4	5	6	7
$I(SZ_{v})$	4.9898	5.7955	6.3707	6.8177	7.1833	7.4926
$I(SZ_{e})$	4.9754	5.7764	6.3524	6.8013	7.1686	7.4793
$I(SZ_{ve})$	4.9839	5.7871	6.3624	6.8101	7.1764	7.4863
$I(PI_{v})$	5.0370	5.8749	6.4646	6.9197	7.2903	7.6029
$I(PI_{e})$	5.0367	5.8748	6.4645	6.9197	7.2903	7.6029

On observing the numerical values, we can infer that the entropy values obtained from $PI_v$ index are higher compared to other indices. Further, these values are directly proportional to size of the molecular framework. Entropy calculations were limited to $p=7$ as the increase in entropy values at higher dimensions becomes negligible. For example, the entropy at $p=67$ is 12.02, while at $p=100$, it is 12.8216, indicating minimal variation only at decimal places. This reveals that the complexity of these networks can be sufficiently captured within lower dimensions primarily because entropy is a natural logarithmic function. Hence it converges rapidly to the asymptotic limit within reasonably large sizes. The following inequality is observed from Table 4, for $x,y \in N$, $2< x < y$ and *G*(*p*) be square dimension framework of FAR, then the inequality holds:

$$\begin{aligned} I(X(G(x))) - I(X(G(x-1))) > I(X(G(y))) - I(X(G(y-1))) \end{aligned}$$

Further, we confirm that the inequality holds for degree and neighborhood degree sum based entropies from literatures^25,38,39^.

### Scaled entropy comparison of zeolites FAR and SOD

**Figure 8:**
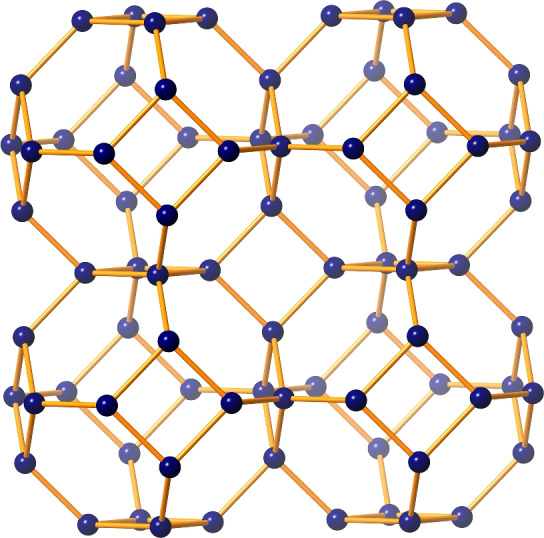
SOD(2).

FAR is a mineral derived from the cancrinite sodalite group and the investigation on distance-based indices of sodalite-type materials (SOD) using the cut method has been already carried out in the article^32^. In this section, we compare the scaled entropies of FAR and SOD frameworks. The scaled entropies of a molecular system are calculated by dividing the entropy measure by the total number of edges in the system as bond-wise topological indices and entropies are considered. The molecular framework of square sodalite is denoted by *SOD*(*p*) and *SOD*(2) is illustrated in Fig. 8.

To find the entropies of SOD-type materials, we follow the same procedure mentioned in “Information entropies of FAR” along with the cuts and their parameters available in the literature^32^. The numerical entropy values of *SOD*(*p*) are tabulated in Table 5.

**Table 5:** Szeged-type entropies of zeolite *SOD*(*p*).

*I*(*X*)	*p* values					
	2	3	4	5	6	7
$I(SZ_{v})$	4.8323	5.5717	6.1070	6.5283	6.8759	7.1719
$I(SZ_{e})$	4.8284	5.5569	6.0897	6.5109	6.8594	7.1564
$I(SZ_{ve})$	4.8308	5.5652	6.0992	6.5204	6.8682	7.1647
$I(PI_{v})$	4.8520	5.6204	6.1738	6.6067	6.9622	7.2640
$I(PI_{e})$	4.8520	5.6204	6.1738	6.6067	6.9622	7.2640

**Figure 9:**
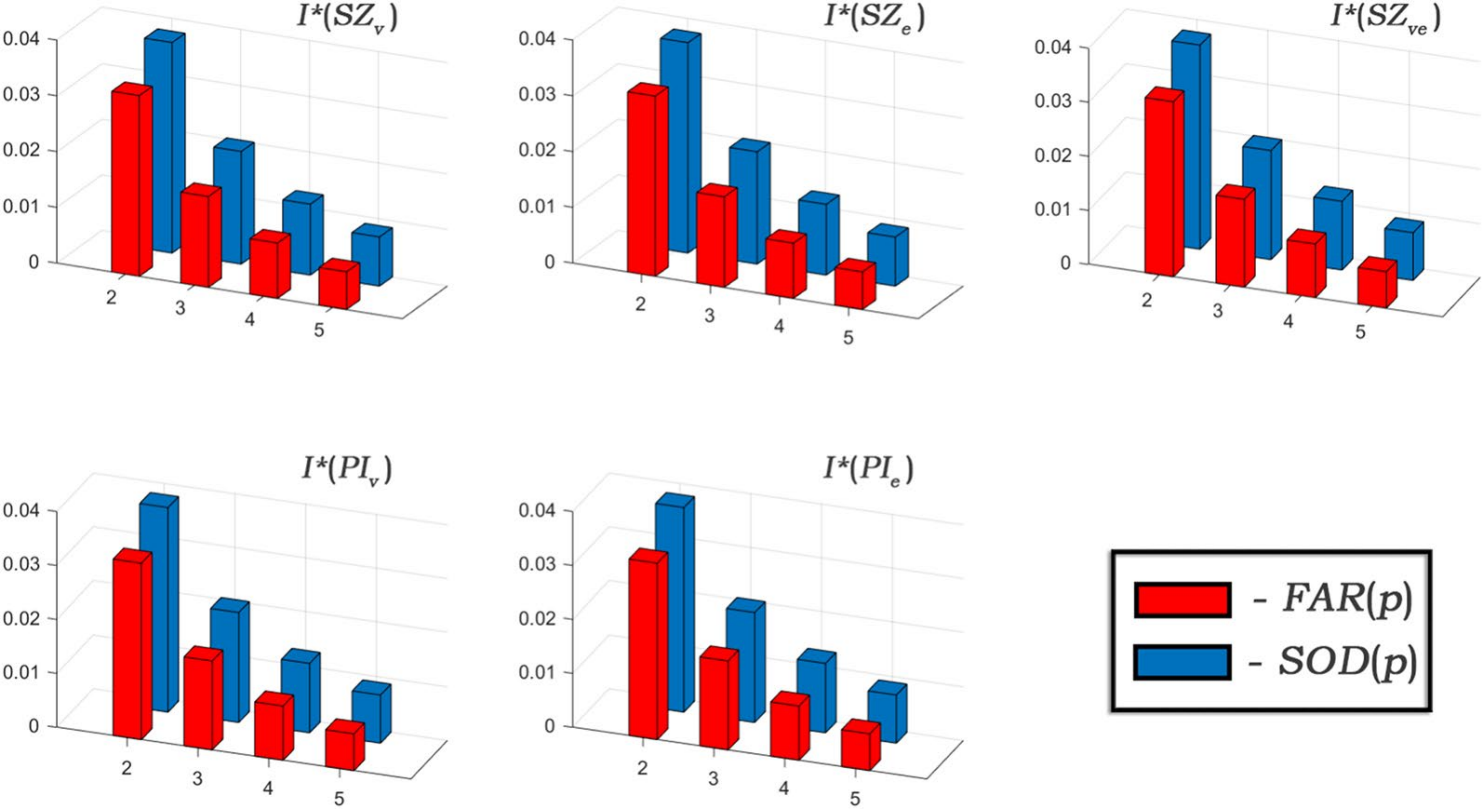
Scaled entropy comparison of FAR and SOD when $2 \le p \le 5$.

In Table 4 and 5, the Padmakar-Ivan entropies obtained from $PI_v$ and $PI_e$ are nearly identical because both indices are formulated using an additive function of closeness measures. This additive formulation inherently minimizes variations in the computed entropy values because entropy is a natural logarithmic function. While the other indices utilize multiplicative functions, which amplify variations, the additive nature of $PI_v$ and $PI_e$ produces more uniform entropy values. Moreover in Table 4, the apparent identical values after $p=4$ are due to rounding to four decimal places in natural logarithmic functions; small differences do exist, such as when $p=5$, where $PI_v$ yields an entropy of 6.9197, while $PI_e$ produces 6.9197 when rounded but differs at higher precision ($PI_v$: 6.91968384984741, $PI_e$: 6.91965131744255). For a comparative analysis, we have provided the scaled entropy measures ($I^*$) of SOD and FAR frameworks in Table 6, additionally it has been graphically illustrated in Fig. 9.

**Table 6:** Scaled entropies of *FAR*(*p*) and *SOD*(*p*).

$I^*(X)$	*FAR*(2)	*SOD*(2)	*FAR*(3)	*SOD*(3)	*FAR*(4)	*SOD*(4)	*FAR*(5)	*SOD*(5)
$I^*(SZ_v)$	0.03240	0.03775	0.01628	0.02019	0.00992	0.01272	0.00674	0.00882
$I^*(SZ_e)$	0.03231	0.03772	0.01623	0.02013	0.00989	0.01269	0.00672	0.00880
$I^*(SZ_{ve})$	0.03236	0.03774	0.01626	0.02016	0.00991	0.01271	0.00673	0.00881
$I^*(PI_v)$	0.03271	0.03791	0.01650	0.02036	0.01007	0.01286	0.00684	0.00893
$I^*(PI_e)$	0.03271	0.03791	0.01650	0.02036	0.01007	0.01286	0.00684	0.00893

Our study confirms that the scaled entropy of the SOD-type framework is higher than the FAR-type. This suggests that the SOD-type of frame work exhibits a greater degree of information disorder compared to the FAR-type zeolite. The information entropies measure the extent of topological disorder. As the FAR framework exhibits a more compact and organized atomic arrangement, where atoms are positioned in closer proximity, the computed topological entropy is lower. In contrast, the SOD framework exhibits a more irregular topology, and in particular greater peripheral irregularity with higher structural complexity, resulting in a greater entropy. These topological variations account for the observed differences in entropy values, reflecting the order/disorder structural characteristics of each framework. Considering the integration of deep and machine learning approaches, we believe that our current study could stimulate advanced topology-driven machine learning algorithms.

## Conclusion

In this article, we have presented techniques to develop topological measures for the complexities of the zeolite FAR-type frameworks by utilizing well-established distance-based topological indices. The generalized expressions to compute these indices have been derived using the standard cut method and the properties of partial cubes. With the aid of these results, we have calculated the Szeged-type entropies of the framework by incorporating Shannon’s information entropy formula. These distance-based indices and the entropies can in future be applied in QSAR predictions. Additionally, we have compared the scaled entropies of FAR and SOD-type frameworks to contrast their information disorders. Our studies reveal that sodalite (SOD) frameworks exhibit greater entropies compared to the farneseite-type frameworks. In the future, researchers can determine Szeged-type entropy measures for various other zeolite variants to understand the complexity of their frameworks. Further, although it may not be feasible to carry out fully ab initio quantum chemical computations on a larger set of FAR networks, it is feasible to study smaller building blocks using density functional theories (DFT). One can then integrate such small-scale DFT computations with machine learning approaches to extrapolate to larger frameworks. Such integrated techniques could enhance the prediction of molecular electronic properties for the FAR zeolite framework.

## Data Availability

Data is provided within the manuscript.
